# The Annual Address to the Candidates for Degrees and Licenses in the Medical Institution of Yale College, February 26, 1839

**Published:** 1839-03-23

**Authors:** 


### BIBLIOGRAPHICAL NOTICES.

*The Annual Address to the Candidates for Degrees and Licenses in the Medical Institution of Yale College, February* 26, 1839. By Thomas Miner, M. D., Member of the Board of Examination, and late President of the Connecticut Medical Society. (Published at the request of the Class.) New Haven: 1839. 8vo. pp. 20.

*Annual Address to the College of Physicians and Surgeons of Lexington; in which the principles and practice of Medical Ethics are illustrated, and urged as essential to the welfare of the profession. Delivered in the Medical Hall January* 1*st*, 1839, By Thomas D. Mitchell, M. D., Professor of Materia Medica and Therapeutics in the Medical department of the Transylvania University; President of the College of Physicians and Surgeons, etc. etc. etc. (Published by request of the College and the Medical Class.) Lexington, Ky: 1839. 8vo. pp. 32.

Dr. Miner's address contains much excellent advice to the young physician, conveyed in a very plain and agreeable manner. Dr. M. thinks and writes well.

Dr. Mitchell has succeeded admirably in compressing most of the cardinal points of medical ethics into a readable size, and has, consequently, conferred a benefit on the profession at large. The Doctor's remarks on Empiricism strike us as conceived in a commendable spirit and happily expressed.

"I know not a more prolific source of mischief in the science of medicine, than *Empiricism*, and none are more inclined to practice it, in some one or other of its manifold forms, than our young graduates. This may be accounted for, in some measure, by the power of example, the tendency common to our nature to be imitators, rather than originals.

By some, the term *Empiricism* has been restricted to the use or countenance of avowed nostrums, and this is undoubtedly a part of the evil. But, in our judgment, every species of random practice, the exhibition of remedial agents, without a rational *why* or *wherefore*, prescribing for names, rather than for symptoms, and every other device resorted to by the practitioner, to save the labour of thinking, may with strict propriety, be placed to the account of Empiricism.

I do not say, that he is an empiric, who employs a popular remedy, that is even lauded in the newspapers, as a panacea, provided he is acquainted with its actual composition, and applies it judiciously, watching its operation, and carefully noting its effects. Under such circumstances, it is lawful to resort to any remedy, and to give it a fair trial, if its action appears to be salutary. This was, virtually, the practice of Sydenham, in regard to every new article that presented itself, as proper for medical use. He made a cautious trial of the remedy, in order to learn its effects, and he persevered in its exhibition, until satisfied. The man who proceeds on such ground, cannot greatly err; and as every remedy, whether it be called a nostrum or by some other name, is new to the physician who has never employed it, he must pursue the plan of Sydenham, or give the article because his neighbour does so, or reject it entirely.

But the case is otherwise, if, without an acquaintance with the constituents of a popular remedy, the physician condescends to administer it, on the testimony of the press, or to gratify the whims of a superstitious patient. Such conduct cannot be defended against the charge of empiricism.

Physicians are often interrogated, touching the value of the patent medicines that are blazoned in our newspapers, as sovereign remedies for all the aches and pains of life; and the good-natured inquirer, half inclined to believe the puffs he reads, is sometimes displeased, if the doctor hesitates to give an opinion, although confessedly a stranger to the composition of the article. To expect a discreet physician to commit himself thus, is about as unwise, and equally ridiculous, as to consult him on the treatment of a sick man a thousand miles off, concerning whose case there is no other information than that he sickened ten years ago, is the son of A. B., born in the year 1800, &c. &c. What physician could refrain from smiling, if consulted on such ground, and how many would treat the affair as a mere hoax? And yet, not a few well-meaning persons seem to think a doctor quite illiberal and narrow-minded, who withholds an expression of approbation from popular remedies, that have done so much service to their friends.

The proprietors of nostrums are often complete monomaniacs; and because they can discern excellencies in a panacea, seem to think that all should laud the praises of their specifics. And they have the presumption to solicit the countenance of medical men, on the faith of the scores of printed certificates, which, in their estimation, are absolutely infallible. They expect the sanction of the profession, too, while they refuse to confide to them the secret of the composition; and, strange to tell, some medical men have so far laid aside their dignity, as to gratify the impudent request. A physician should not prescribe, nor patronise, in any way, a medicine, the ingredients of which are unknown except to the proprietor.

It sometimes occurs, that empiricism clandestinely supplants the efforts of the regular practitioner, and occasions not a little embarrassment. Our best patients, labouring under chronic diseases, are occasionally beguiled to try some vaunted mixture, that has the reputation of infallibility; and if temporary relief be procured, they sometimes venture to rebuke their long-tried and faithful physician, for his want of discrimination. However vexatious such occurrences may be, it is, sometimes, wise to bear with them, and to maintain our friendly relation, as though nothing of the kind had transpired. I have watched such cases with some attention, and have witnessed the return of confidence, as the necessary result of dissatisfaction or disgust with the pretended specific; and in this way, empiricism has been more effectually counteracted, than it could have been by the direct assault of the physician. As a general rule, however, we should frown upon, and, if possible, frown down every *secret* remedy, the chief recommendation of which is, that nobody but the proprietor understands its composition.

But there is another sort of empiricism, that is exceedingly fashionable, and therefore the more formidable; I mean, the quackery of applying any given mode cf treatment to a disease, irrespective of the varying conditions of the system. There are even yet many physicians who prescribe for names instead of things, although the venerable Rush, in his day, was accused of slandering the profession, when he charged them with this kind of mal-practice. Such practitioners are mere routinists. They have one pill for this and another for that case, and whenever you meet them, the well known pill box is forthcoming, and the entire treatment can be anticipated, almost with unerring certainty. They regard disease as possessed of characters, unvarying as the granite and feldspar, the tiger and the lion; and hence their therapeutics make no account of the influence of season and other controlling circumstances, that so often modify and aggravate disease.

The intelligent physician must needs be a student as long as he occupies the field. He knows that the forms of morbid action are continually changing, and that his constant vigilance is demanded, to meet the exigencies that surround him; and he regards as preposterous, all curative efforts, that are not based on this fundamental principle. With the mere mechanic in medicine, who looks upon every shade of disease, as a distinct piece of statuary, moulded by the chisel, he has no sympathy. He professes allegiance to a system, that has sound philosophy for its basis, and that demands the daily exercise of the ablest minds.

It is a mistake to identify empiricism with the ungraduated practitioner. Some of the most flagrant instances of quackery that have ever fallen under my observation, were in the ranks of medical diplomatists; while, on the other hand, I have witnessed an enlightened and honorable course, more than once, in those who never sought the distinction of a degree in medicine. A judge in Pennsylvania, who was highly incensed at the stupidity of a court officer, recently appointed by the governor, observed, in the hearing of the officer, "the governor can make prothonotaries and clerks, but he can't give them brains." In like manner we may affirm, that a school of medicine may confer the Doctorate on the undeserving and unqualified, but that act does not infuse into the party, either the powers of investigation, or plain common sense, and much less straightforward honesty.

With all his characteristic mildness, Dr. Rush boldly denounced a class of men, to whom he gave the appellation of "traders in medicine," more than thirty years ago. And these persons were not quacks, in the ordinary acceptation of the term; but actual graduates, legally invested with the honors of the Doctorate. Though destitute of the spirit of honorable enterprise, they had foisted themselves upon colleges, with scarcely any sort of preparation, and by some indefinable process, had squeezed through the formalities of the *green-room.* Strangers to the finer feelings of human nature, they took up with the profession of medicine, just as the tinker falls on his avocation, viz., for the purpose of making the most money, in what appeared to be the easiest way. These are the SHYLOCKS of the profession, who want the pound of flesh and mean to get it. You might as well essay to talk the tornado into silence, as to seek the reformation of such men by argument. They are utterly incorrigible. The voice of persuasion falls on their ear as powerless as on the thundering, senseless cataract. The effort is abortive from the very nature of the case. Where then is our hope? We reply, in the rising generation. Let the proper training be given to all our young men, before they are permitted to commence the study of medicine, and let them enjoy the instruction of physicians whose moral sense is not less vigorous than their ability to teach, and who rightly appreciate the dignity of their station. If this discipline fail to resuscitate the decaying energies of the profession, we must lie down in despair, greeted only with the appalling inscription glaring upon the vision from every quarter, "the glory has departed."

Let the young graduate who would rise superior to the trickery of empiricism, resolve to do every thing in his profession by rule, and according to just principles drawn from perpetual investigation of disease, and all the passing events that may modify the type and grade of morbid action. Setting out with this determination, he will soon acquire a fondness for the course; and in a few years the habit will be so fixed, that to practise on any other basis would be a violation of his nature."

